# Detection of Alzheimer’s disease by displacement field and machine learning

**DOI:** 10.7717/peerj.1251

**Published:** 2015-09-17

**Authors:** Yudong Zhang, Shuihua Wang

**Affiliations:** 1 School of Computer Science and Technology, Nanjing Normal University, Nanjing, Jiangsu, China; 2 School of Electronic Science and Engineering, Nanjing University, Nanjing, Jiangsu, China; 3 Jiangsu Key Laboratory of 3D Printing Equipment and Manufacturing, Nanjing, Jiangsu, China

**Keywords:** Region detection, Machine vision, Generalized eigenvalue proximal SVM, Alzheimer’s disease, Whole brain analysis, Support vector machine (SVM), Machine learning, Twin SVM (TSVM)

## Abstract

**Aim.** Alzheimer’s disease (AD) is a chronic neurodegenerative disease. Recently, computer scientists have developed various methods for early detection based on computer vision and machine learning techniques.

**Method.** In this study, we proposed a novel AD detection method by displacement field (DF) estimation between a normal brain and an AD brain. The DF was treated as the AD-related features, reduced by principal component analysis (PCA), and finally fed into three classifiers: support vector machine (SVM), generalized eigenvalue proximal SVM (GEPSVM), and twin SVM (TSVM). The 10-fold cross validation repeated 50 times.

**Results.** The results showed the “DF + PCA + TSVM” achieved the accuracy of 92.75 ± 1.77, sensitivity of 90.56 ± 1.15, specificity of 93.37 ± 2.05, and precision of 79.61 ± 2.21. This result is better than or comparable with not only the other proposed two methods, but also ten state-of-the-art methods. Besides, our method discovers the AD is related to following brain regions disclosed in recent publications: Angular Gyrus, Anterior Cingulate, Cingulate Gyrus, Culmen, Cuneus, Fusiform Gyrus, Inferior Frontal Gyrus, Inferior Occipital Gyrus, Inferior Parietal Lobule, Inferior Semi-Lunar Lobule, Inferior Temporal Gyrus, Insula, Lateral Ventricle, Lingual Gyrus, Medial Frontal Gyrus, Middle Frontal Gyrus, Middle Occipital Gyrus, Middle Temporal Gyrus, Paracentral Lobule, Parahippocampal Gyrus, Postcentral Gyrus, Posterior Cingulate, Precentral Gyrus, Precuneus, Sub-Gyral, Superior Parietal Lobule, Superior Temporal Gyrus, Supramarginal Gyrus, and Uncus.

**Conclusion.** The displacement filed is effective in detection of AD and related brain-regions.

## Introduction

Alzheimer’s disease (AD) is an abnormal process of aging. AD is a special category of senile dementia (SD) which leads to short-term and long-term memory, thinking, and behavior (Dong et al., 2015a). Research on AD has attracted scholars from all over the world because of its importance and effect on the society. Symptoms of AD may become severe enough to interfere with daily life, and to death (Goh et al., 2014; Hahn et al., 2013). There is neither cure nor treatment for AD. In 2006, more than twenty million people in the world suffered from this disease (Murphy et al., 2011). AD is predicted to influence 1 in every 85 people worldwide about thirty years later, and more than forty percent of prevalent cases need high level of care (Brookmeyer et al., 2007).

Now that the earth is growing into an aging society, AD has caused heavier burdens on families and society than before (Esposito et al., 2013; Kantanen et al., 2014). In China, AD accounts for more than half of SD, which extracts a total economic loss of more than eighty billion yuan every year, and is responsible for nearly sixty billion yuan in healthcare costs every year (Song & Wang, 2010). In United States, healthcare on people with Alzheimer’s disease currently costs roughly $100 billion per year and is predicted to cost $1 trillion per year by 2050 (Miller, Erlien & Piersol, 2012; Yuan, Machado & Arias-Carrion, 2014).

Nowadays, it is beneficial to develop early and accurate detection methods for AD, which is also necessary for the treatment and management for controlling the deterioration of AD (Zhang et al., 2015b). A 3D scan of the whole brain becomes acceptable and affordable with recent advances in neuroimaging technology (Zhang et al., 2015e; Zhang et al., 2014b), especially by the help of the most popular imaging technique: magnetic resonance imaging (MRI). With its high-resolution magnetic resonance (MR) images, the diagnostic accuracy of AD are greatly enhanced. MR images already play an essential role in detecting AD from normal elder controls (NC).

In this study, we proposed to employ displacement field (DF) to track the morphometry from normal brains to AD brains. The main advantage is that this method does not need to segment region-of-interest (ROI) beforehand, namely, it obtains the DF for the whole image. The classical level-set method was employed to estimate the DF.

This work is structured as follows: ‘State-of-the-Art’ provides the state-of-the-art. ‘Materials and Methods’ presents the materials and methodology. ‘Experiments and Results’ gives the experimental results. ‘Discussion’ discusses the results and the proposed method. ‘Conclusions’ concludes this work. The nomenclature in this work is listed in Table 7.

## State-of-the-Art

In the past, most diagnosis work was carried out by measuring region of interest (ROI) of brain MR images, since researchers already know several typical AD-related regions and corresponding shape deformation (Anagnostopoulos et al., 2013; Kubota, Ushijima & Nishimura, 2006), such as the enlarged ventricles, the shrinkage of hippocampus (HC), and the shrinkage of cortex (Pennanen et al., 2004). Somehow, the ROI-based methods suffer from some shortcomings: (i) The ROI methods need *a priori* information and expert knowledge. (ii) The detection accuracy relies on the experiences of the interpreters (Yang et al., 2015). (iii) The mutual information among the voxels is difficult to implement (Lee, Park & Han, 2013; Xinyun, Wenlu & Xudong, 2011). (iv) We need to explore other potential regions that may be connected to AD (El-Dahshan et al., 2014). (v) Automatic segmentation of ROI is not feasible in practice, and examiners tend to segment the brain manually (Zhang et al., 2013).

Recently, a new type of methods, the “*whole-brain analysis*”, gets popularity since it considers all voxels in the brain as a whole. It does not need to segment the brain beforehand, and it does not need any biomarker for classification task. The main disadvantage is the curse of dimensionality, which can be solved as the high-speed computers are very cheap nowadays (Álvarez et al., 2009). The whole-brain analysis heavily relies on pure computation, and it can be accomplished by only computer scientists, after physicians help to label the data as either AD or healthy. Generally, the whole-brain analysis treats the whole brain as a ROI, and it consists of two stages: feature extraction and classification. We reviewed more than 10 literatures, and analyzed them in detail.

Scholars have presented various methods to extract efficient features for AD and other types of pathological brain detection.^1^1Some abbreviations are modified to avoid conflict within this paper. In addition, various classification models and methods exist, nevertheless, only a few of them are suitable for MR images. El-Dahshan, Hosny & Salem (2010) extracted the approximation and detail coefficients of 3-level discrete wavelet transform (DWT). They used artificial neural network (ANN) and K-nearest neighbors (KNN) classifiers. Plant et al. (2010) used brain region cluster (BRC). They suggested to use information gain (IG) to evaluate the interestingness of a voxel, and applied clustering algorithm to identify groups of adjacent voxels with a high discriminatory power. They used support vector machine, Bayes statistics, and voting feature intervals (VFI), with the aim of pattern classification. Park (2012) employed manifold learning for classification. Their use of the first and second distance measure resulted in an 18% and 46% error rate for classifying between patients with AD and normal patients, respectively. Chaves et al. (2012) utilized large margin-based methodology for AD detection in SPECT and PET images. Their system yielded accuracy, sensitivity and specificity values of 90.67%, 88% and 93.33% (for PET) and 92.78%, 91.07% and 95.12% (for SPECT), respectively. Saritha, Joseph & Mathew (2013) was the first to use wavelet-entropy in pathological brain detection. They employed spider-web plots to reduce the size of feature space. They also used the probabilistic NN (PNN) for classification. Zhang et al. (2015a) suggested that removing spider-web-plot yielded the same classification performance. Savio & Grana (2013) presented a novel method that employed deformation-based morphometry (DBM) method. They tested five features, and found three features performed excellent as trace of Jacobian matrix (TJM), modulated GM (MGM), and Geodesic Anisotropy (GEODAN). Furthermore, they utilized Pearson’s correlation (PEC), Welch’s *t*-test (WTT), and Bhattacharyya distance (BD), to measure the significance of voxel site. Kalbkhani, Shayesteh & Zali-Vargahan (2013) modeled the detail coefficients of 2-level DWT by generalizing autoregressive conditional heteroscedasticity (GARCH) statistical model, and the parameters of GARCH model were considered as the primary feature vector. They tested the KNN and SVM models. Wang et al. (2014) proposed to utilize the undersampling (US) technique on a three-dimensional image. They used singular value decomposition (SVD) to select features. Finally, they combined kernel SVM (KSVM) with decision tree (KSVM-DT). Zhou et al. (2015) followed Saritha’s method, and again employed wavelet-entropy for feature extraction. Naive Bayes classifier (NBC) was employed for abnormal brain detection. Harikumar & Kumar (2015) analyzed the performance of ANN, in terms of classification of medical images, using wavelets as feature extractor. Their classification accuracy achieved 96%. Zhang et al. (2015c) employed discrete wavelet packet transform (DWPT). They utilized Tsallis entropy to get wavelet packet entropy features. They introduced a generalized eigenvalue proximal support vector machine (GEPSVM). Nazir, Wahid & Khan (2015) suggested to use filters for the removal of noises, and extracted color moments as mean features. Finally, they achieved an overall accuracy of 91.8%. Dong et al. (2015b) employed the eigen-brain (EB) to extract features, and then they employed Welch’s t-Test (WTT) for the aim of reducing features. They proposed to use SVM with radial basis function (RBF). Damodharan & Raghavan (2015) combined tissue segmentation and neural network for brain tumor detection. Zhang et al. (2015f) proposed a novel classification system that implemented 3D discrete wavelet transform (3D-DWT) to extract wavelet coefficients the volumetric image.

After reviewing the latest literature, two findings can be obtained: (1) The DWT based features are efficient. However, we presented a novel feature of displacement field, which is the first to be used in MR images. (2) SVMs were commonly used, compared with conventional decision tree, artificial neural network (Wang et al., 2015a; Zhang et al., 2015d), and other classifiers (Collins & Pape, 2011). Hence, we continue to use SVM. Besides, two variants of SVM are introduced in this study: generalized eigenvalue proximal SVM (GEPSVM) and twin SVM (TSVM), with the aim of augmenting the classification performance further.

## Materials and Methods

### Materials

The open dataset was downloaded from “Open Access Series of Imaging Studies (OASIS)” (Ardekani, Figarsky & Sidtis, 2013), which consists of 416 subjects aged from eighteen to ninety-six. All subjects are right-handed. Then, we merely selected 126 samples (28 ADs and 98 NCs) from the dataset. The demographic statuses are reported in Table 1. Following common convention, clinical dementia rating (CDR) was interpreted as the target (label). Note that subjects either with missing records or under sixty years old were removed.

**Table 1:** Demographic status of subjects. MMSE denotes mini-mental state examination.

Characteristic	Alzheimer’s disease	Normal control
Subject #	28	98
Age	77.75 ± 6.99	75.91 ± 8.98
Gender (M/F)	9/19	26/72
Education	2.57 ± 1.31	3.26 ± 1.31
Socioeconomic status	2.87 ± 1.29	2.51 ± 1.09
MMSE	21.67 ± 3.75	28.95 ± 1.20
CDR	1	0

### Co-registration and brain-masking

All three-dimensional MR brain images of each subject were motion-corrected, and coregistered to generate an averaged three-dimensional image, with the aim of increasing signal-to-noise ratio. Afterwards, those three-dimensional images were spatially co-registered to the Talairach space and then were brain-masked.

**Figure 1:**
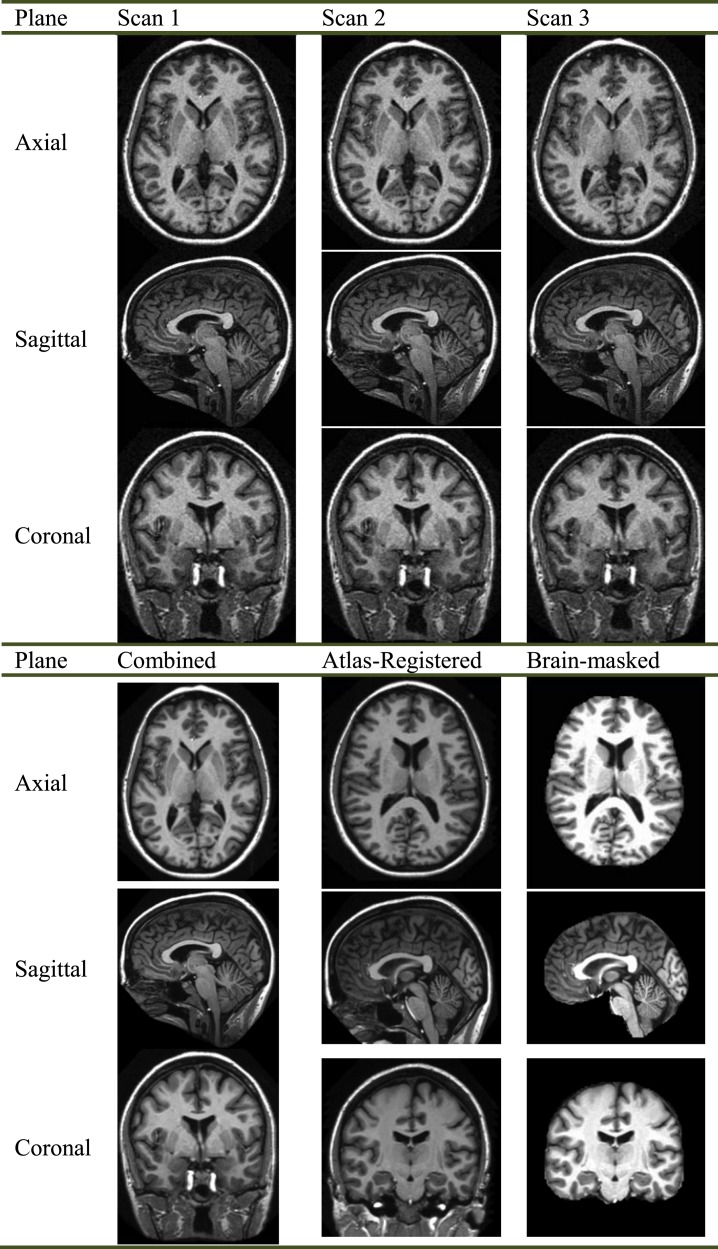
Preprocessing of a specified subject.

Figure 1 offers an example of the preprocessing on 3D images with resolution of 1 mm × 1 mm × 1.25 mm. The motion-correction procedure registered the images of three scans, and then generated an image in the original acquisition space with resampling to 1 mm × 1 mm × 1 mm. Finally, the averaged image was normalized to the Talairach coordinate space, and brain-extracted (Fig. 1).

### Key slice selection

Calculating the displacement field on the whole brain was time-consuming. Therefore, we introduced the “key-slice (KS) selection” method that picks up key slices containing structures indicative of AD from NC. The procedure was as follows: we established a criterion called “inter-class variance (ICV)” *v* as

(1) \begin{eqnarray*} v(k)=\Vert {\mu }_{\text{A}}(k)-{\mu }_{\text{N}}(k)\Vert ^{2} \end{eqnarray*}

where *k* is the index of key slice, ‖ ⋅ ‖^2^ represents the *l*_2_-norm, *μ*_A_ and *μ*_N_ represents the mean of gray-level values of the *k*th slice of AD subjects and NC subjects, respectively, and *v* denotes the ICV. We select the KSs whose ICVs are larger than half of maximum ICV, with 10× undersampling factor (i.e., every ten slices).

**Figure 2:**
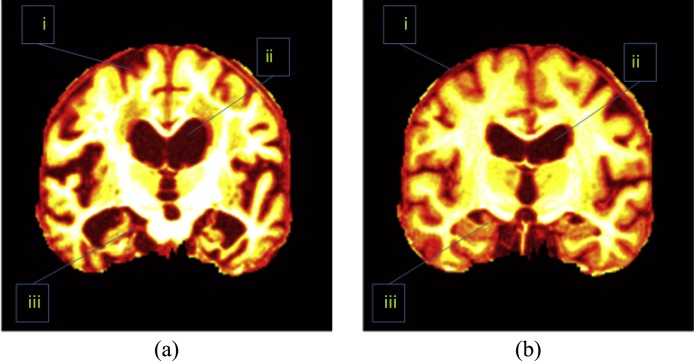
Important regions between (A) an AD brain and (B) a normal brain. i, CC; ii, ventricle; iii, HC. (Pseudocolor enhancement is performed for enlarging contrast.)

Besides, the direction of key slice can be either sagittal, or axial, or coronal. Scholars found images along coronal direction give a well-defined view compared to those along sagittal and axial directions. Figure 2 shows an image along coronal direction has an advantage because it covers 3 most important AD-related regions, which are treated as indicative of AD. These AD-related regions consist of the cerebral cortex (CC), the HC, and the ventricle. When sagittal or axial directions are used, two or even more slices are needed to cover those tissues. Therefore, we chose the coronal direction for key slice selection, in order to use less slices.

### Shape registration

**Figure 3:**
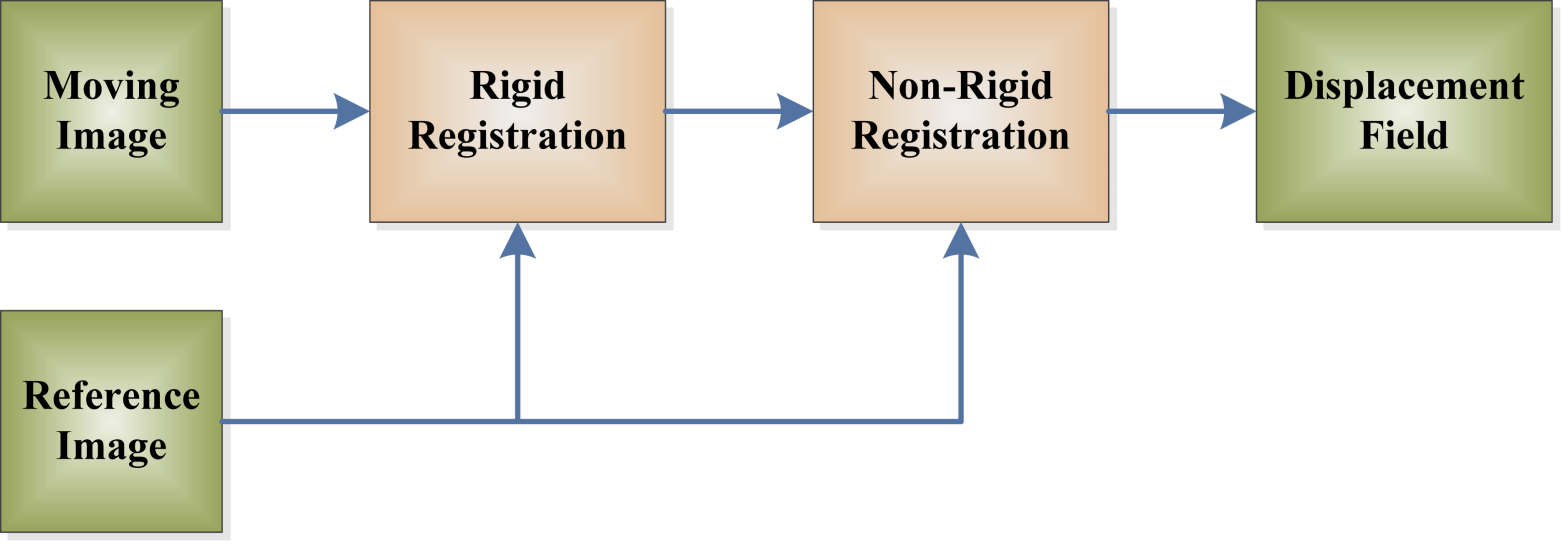
Flowchart of displacement field.

The shape registration consists of both global and local registrations. The global registration estimates the rigid parameters, which was accomplished in ‘Co-registration and brain-masking’. Then, the local registration finds the “displacement field (DF)” between the moving and reference images (see Fig. 3).

The rigid registration is an essential preprocessing procedure before non-rigid registration. It can fix the deformation stemming from the position, move, and pose of patients. Then, the following non-rigid registration can reflect the shape deformation of diseases.

The problem of finding the non-rigid estimation can be thought of a motion estimation task between a normal brain (moving) and an AD brain (reference). Several types of solutions are available to solve this task, such as spline function based methods, phase-correlation methods, fluid methods, optical-flow methods, elastic methods, etc. Among the above methods, the first type is parametric, hence, this kind of solution needs to solve optimal spline-based function parameters (Petibon et al., 2013). The second type needs a mass of computation resources; it is difficult to determine the local search range, and it is impossible to guarantee to find global optimal points (Lu, Yang & Zhang, 2014). The other three methods are non-parametric. They find the DF by solving directly a predefined physical model using partial differential equation (PDE) (Yang et al., 2007).

The level-set motion estimation method (Huang et al., 2014; Lee, Sandhu & Tannenbaum, 2013; Vandemeulebroucke et al., 2012) is a rather novel method, which is formed on the basis of the level set evolution theory. The moving image *I*_1_ morphs iteratively along its gradient direction, till it deforms close to the given reference image *I*_2_. The displacement field is in the form of

(2) \begin{eqnarray*} \frac{\text{d}V}{\text{d}t}=\left({I}_{2}-{I}_{1}(V)\right)\frac{\nabla {I}_{1}(V)}{\left\vert \nabla {I}_{1}(V)\right\vert } \end{eqnarray*}

where *V* represents the displacement field, and *I*_1_(*V*) the deformed image of *I*_1_ by *V*. The above equation can be solved by iterative algorithms described in reference (Kodipaka et al., 2007).

**Figure 4:**
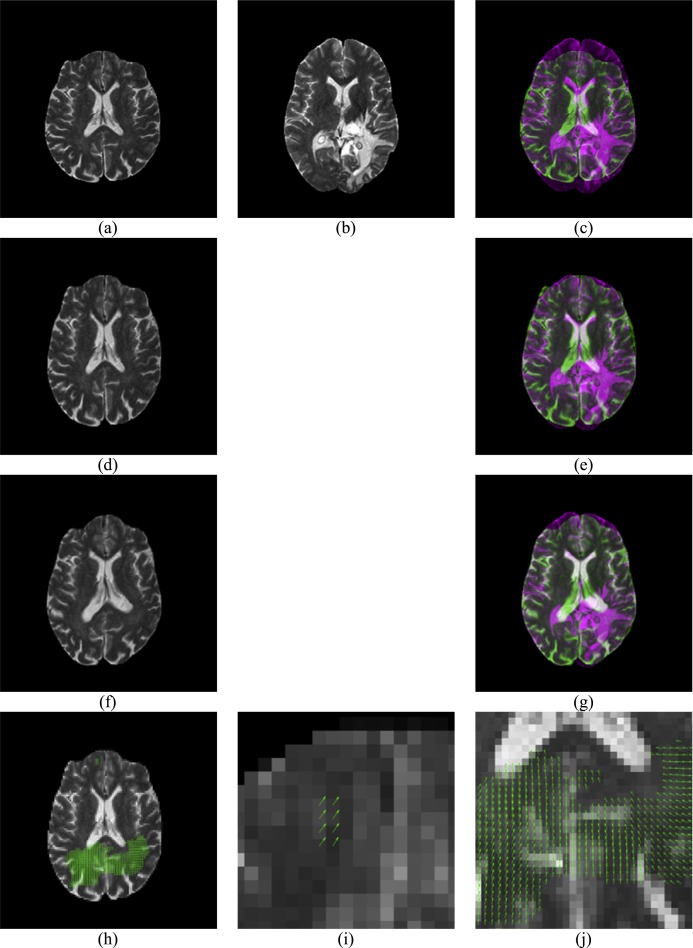
Illustration of displacement field between a Glioma brain and a normal one. (A) Moving Image *I*_1_; (B) Reference Image *I*_2_; (C) Overlap of (B) and (A); (D) Rigid Registration of *I*_1_; (E) Overlap of (B) and (D); (F) Non-rigid registration of (D); (G) Overlap of (B) and (F); (H) Displacement Field between (B) and (D); (I) Enlarged CC of (H); (J) Enlarged temporal and occipital lobe of (H).

Figure 4 gives an example of the displacement field between a glioma brain and a normal one. Figure 4A presents a moving image while Fig. 4B is a reference image. Their overlap is shown in Fig. 4C. Apparently, the reference image is larger than the moving image, and they are not quite a match. Figure 4D shows the result of rigid-registration, and Fig. 4E shows the two images now match well except the cortex, the temporal and occipital lobe. Figure 4F shows the result of non-rigid registration. Compare to Fig. 4B, they closely match each other, and Fig. 4G also suggests a nearly-perfect match. The displacement field is shown in Fig. 4H, with two enlarged areas in Figs. 4I and 4J.

The values of displacement field are complex, i.e., the real part represents the displacement field along the horizontal direction, and the imaginary part along the vertical direction. Those values of Cartesian system are transformed into polar coordinate system, viz., the magnitude-direction spaces. Hence, the inputs to the classifiers are two fold in this study: (1) the directions of the displacement vector of the whole brain and (2) the magnitude of the displacement field of the whole brain. We did not use the sign of the displacement vector, which were commonly used in literature (Das Gupta & Tamadapu, 2013; Kodipaka et al., 2007). The reason is the direction field already contains the information of sign of the displacement.

### Method of region detection

Here, we proposed a visual interpretation approach based on displacement field to detect regions **R** that can distinguish AD and NC.

(3) \begin{eqnarray*} \mathbf{R}=\{ (x,y)\vert V(x,y)\vert > T\} . \end{eqnarray*}

Here | ⋅ | represents the magnitude, *V*(*x*, *y*) represents the displacement field at point of (*x*, *y*), *T* the threshold. From another point of view, Eq. (3) means we preserve the points whose displacement magnitude is larger than *T*. In this work, *T* is assigned with a value of 5, which is defined empirically. A smaller threshold *T* may introduce more noises in the estimated displacement field; whereas, a larger threshold *T* will drop realistic deformation with short deformation magnitude. Hence, we believe the deformation with magnitude larger than 5 represents realistic deformation in the brain. In all, the visual interpretation is a three-step process (see Table 2).

**Table 2:** Pseudocode of the region detection method.

Region detection	
Step 1	Select a normal brain (*I*_1_) and an AD brain (*I*_2_) from the dataset
Step 2	For each key slice *k*
	Implement level-set displacement-field estimation between *I*_1_ and *I*_2_, and obtain the displacement field *V*.
	Move the points (*x*, *y*), that satisfies |*V*(*x*, *y*)| > 5, to the set **R**.
	End
Step 3	Output **R**.

### Non-parallel support vector machine

Among all recent classifiers, the support vector machine (SVM) has gained popularity as the most excellent classifiers in small-size problem (Zhang & Wu, 2012). To further enhance the classification performance, two new variants of SVM were introduced:

#### Generalized eigenvalue proximal SVM

Original SVM has a limitation that two hyperplanes should be parallel (Wang et al., 2014). Mangasarian & Wild (2006) designed a generalized eigenvalue proximal SVM (GEPSVM). It drops the parallelism restrain on the two planes (remember the parallelism is necessary in original SVM), and requires each hyperplane should be as close as possible to one of the data sets and as far as possible from the other. The latest literature shows that GEPSVM yielded superior performance to canonical support vector machines in terms of sensitivity, specificity, precision, and accuracy (Khemchandani, Karpatne & Chandra, 2011; Shao et al., 2013).

Suppose samples are from either class 1 (denote by symbol *X*_1_) or class 2 (denoted by symbol *X*_2_), respectively. The GEPSVM finds the two optimal nonparallel planes with the form of (**w** and *b* denotes the weight and bias of the classifier, respectively)

(4) \begin{eqnarray*} {\mathbf{w}}_{1}^{T}x-{b}_{1}=0\hspace{1em}\text{and}\hspace{1em}{\mathbf{w}}_{2}^{T}x-{b}_{2}=0. \end{eqnarray*}

To obtain the first plane, we deduce from Eq. (4) and get the following solution

(5) \begin{eqnarray*} ({\mathbf{w}}_{1},{b}_{1})=\mathop{\arg \min }\limits _{(\mathbf{w},b)\not = 0}\frac{\Vert {\mathbf{w}}^{T}{X}_{1}-{o}^{T}b\Vert ^{2}/\Vert z\Vert ^{2}}{\Vert {\mathbf{w}}^{T}{X}_{2}-{o}^{T}b\Vert ^{2}/\Vert z\Vert ^{2}} \end{eqnarray*}

(6) \begin{eqnarray*} \mathbf{U}\leftarrow \left[\begin{array}{c} \displaystyle \mathbf{w}\\ \displaystyle b \end{array}\right] \end{eqnarray*}

where *o* is a vector of ones with any possible dimension according to the context. Simplifying formula (5) gives

(7) \begin{eqnarray*} \min _{(\mathbf{w},b)\not = 0}\frac{\Vert {\mathbf{w}}^{T}{X}_{1}-{o}^{T}b\Vert ^{2}}{\Vert {\mathbf{w}}^{T}{X}_{2}-{o}^{T}b\Vert ^{2}}. \end{eqnarray*}

We include the Tikhonov regularization term to decrease the norm of the variables **U** that corresponds to the first hyperplane in (4).

(8) \begin{eqnarray*} \min _{(\mathbf{w},b)\not = 0}\frac{\Vert {\mathbf{w}}^{T}{X}_{1}-{o}^{T}b\Vert ^{2}+t\Vert \mathbf{U}\Vert ^{2}}{\Vert {\mathbf{w}}^{T}{X}_{2}-{o}^{T}b\Vert ^{2}} \end{eqnarray*}

where *t* is a positive (or zero) Tikhonov factor. Formula (8) turns to the “Rayleigh Quotient (RQ)” in the following form of

(9) \begin{eqnarray*} {\mathbf{U}}_{1}=\mathop{\arg \min }\limits _{\mathbf{U}\not = 0}\frac{{\mathbf{U}}^{T}\mathbf{PU}}{{\mathbf{U}}^{T}\mathbf{QU}} \end{eqnarray*}

where **P** and **Q** are symmetric matrices in size of (*p* + 1) × (*p* + 1)

(10) \begin{eqnarray*} \mathbf{P}\hspace{0.167em} \mathop{=}\limits ^{d e f}\hspace{0.167em} {\left[\begin{array}{cc} \displaystyle {X}_{1}&\displaystyle -o \end{array}\right]}^{T}\left[\begin{array}{cc} \displaystyle {X}_{1}&\displaystyle -o \end{array}\right]+t I \end{eqnarray*}

(11) \begin{eqnarray*} \mathbf{Q}\hspace{0.167em} \mathop{=}\limits ^{d e f}\hspace{0.167em} {\left[\begin{array}{cc} \displaystyle {X}_{2}&\displaystyle -o \end{array}\right]}^{T}\left[\begin{array}{cc} \displaystyle {X}_{2}&\displaystyle -o \end{array}\right]. \end{eqnarray*}

Considering the stationarity and boundedness characteristics of RQ, the answer of (9) is deduced by figuring out a generalized eigenvalue problem (GEP) as

(12) \begin{eqnarray*} \mathbf{PU}=\lambda \mathbf{QU},\hspace{1em}\mathbf{U}\not = 0 \end{eqnarray*}

here the global optimal result of (9) can be obtained at an eigenvector **U**_1_ associated to the smallest eigenvalue *λ*_min_ of formula (12). Therefore, **w**_1_ and *b*_1_ can be deduced from formula (6), and utilized to from the plane as written in formula (4). Afterwards, a similar optimization problem is generated that is analogous to (7) by exchanging the symbols of *X*_1_ and *X*_2_. The eigenvector **U**_2_∗ corresponding to the smallest eigenvalue of the 2nd GEP will obtain the 2nd hyperplane approximate to samples of class 2.

#### Twin support vector machine

Jayadeva & Chandra (2007) provided a novel variant of standard SVM: the twin support vector machine (TSVM). The TSVM is similar to GEPSVM in the way that both obtain non-parallel hyperplanes. The difference lies in that GEPSVM and TSVM are formulated entirely differently. Each of the two quadratic programming (QP) problems in TSVM pair is formulated as a typical SVM. Reports have shown that TSVM is better than both SVM and GEPSVM (Nasiri, Charkari & Mozafari, 2014; Shao et al., 2014; Xu, Qi & Zhang, 2014). Mathematically, the TSVM is constructed by solving the two QP problems

(13) \begin{eqnarray*} \begin{array}{ll} \displaystyle \min _{\text{w}_{\text{1}},{b}_{1},q}&\displaystyle \frac{1}{2}{\left({X}_{1}{\mathbf{w}}_{1}+{o}_{1}{b}_{1}\right)}^{T}\left({X}_{1}{\mathbf{w}}_{1}+{o}_{1}{b}_{1}\right)+{c}_{1}{o}_{2}^{T}q\\ \displaystyle &\displaystyle \text{s.t. }-\left({X}_{2}{\mathbf{w}}_{1}+{o}_{2}{b}_{1}\right)+q\geqslant {o}_{2},q\geqslant 0 \end{array} \end{eqnarray*}

(14) \begin{eqnarray*} \begin{array}{ll} \displaystyle \min _{\text{w}_{2},{b}_{2},q}&\displaystyle \frac{1}{2}{\left({X}_{2}{\mathbf{w}}_{2}+{o}_{2}{b}_{2}\right)}^{T}\left({X}_{2}{\mathbf{w}}_{2}+{o}_{2}{b}_{2}\right)+{c}_{2}{o}_{1}^{T}q\\ \displaystyle &\displaystyle \text{s.t. }-\left({X}_{1}{\mathbf{w}}_{2}+{o}_{1}{b}_{2}\right)+q\geqslant {o}_{1},q\geqslant 0 \end{array} \end{eqnarray*}

here *c_i_* (*i* = 1, 2) are positive parameters, and *o_i_* (*i* = 1, 2) is the same as in formula (5). By this means, the TSVM constructed two hyperplanes. The first term in equations of (13) (14) is the sum of squared distances from the hyperplane to one class. The second term is the sum of error variables. Therefore, minimizing Eqs. (13) and (14) will force the hyperplanes approximate to data of each class, and minimize the misclassification rate. Finally, the constraint requires the plane to be at a distance of more than 1 from data in the other class. Another advantage of TSVM is that its convergence rate is four times faster than conventional SVM (Jayadeva & Chandra, 2007).

### Statistical analysis

Generalization error was obtained by K-fold CV, and K was set to 10 because of two reasons: (1) to balance between computational cost and reliable estimates, and (2) for fair comparison since the common convention is to set K with the value of 10 (Zhang et al., 2014a).

**Figure 5:**
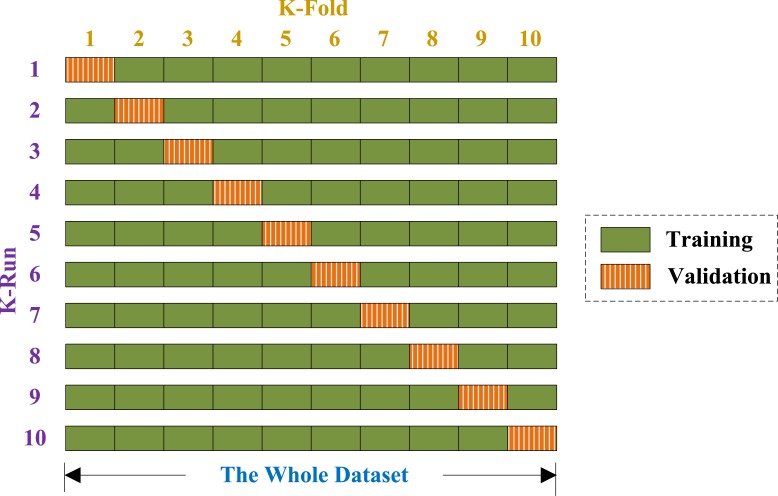
Diagram of a 10-fold cross validation.

For a 10-fold CV, the dataset is randomly divided into 10 mutually exclusively folds of nearly equal size. In each run, 9 subsets are used for training, and the rest for validation (see Fig. 5). Above procedure repeats 10 runs, such that each subset is used once for validation. The 10 results over validation set are combined together along the diagonal blocks in Fig. 5, with the aim of producing an individual out-of-sample evaluation. The 10-fold cross validation repeated 50 times, viz., a 50×10-fold cross validation was implemented.

### Evaluation

We used four indicators to measure which algorithm performed the best. Those four indicators consisted of sensitivity (recall), specificity, accuracy, and precision (Table 3). In this work, a correctly detected AD was treated as TP. After the 50×10-fold cross validation, the final evaluation results are written in the form of “mean ± standard deviation (SD)”.

**Table 3:** Evaluation indicators.

Indicator	Explanation
TP	True Positive
FP	False Positive
TN	True Negative
FN	False Negative
Sensitivity (recall)	TP/(FN + TP)
Specificity	TN/(FP + TN)
Accuracy	(TN + TP)/(FN + FP + TN + TP)
Precision	TP/(TP + FP)

### The whole proposed system

Remember that our aim contains two purposes. First, we need to develop a computer-aided diagnosis (CAD) system and report its performance. Second, we need to locate discriminant voxels that can detect AD from NC subjects; and (ii) The pseudocode is listed in Table 4.

**Table 4:** Pseudocode of proposed method.

Algorithm: proposed method	
Step A	Input the ground-truth imaging data together with their labels.
Step B	Co-registration to Talairach Coordinate by Rigid Registration.
Step C	Pick up the key-slices by ICV (more than half of maximum), with 10× undersampling factor.
Step D	Produce displacement field for each key slice for each subject.
Step E	Submit the displacement field to the classifiers.
Step F	Report the classification performance based on a 50×10-fold cross validation.
Step G	Report the AD-related regions with the points whose displacement magnitude is larger than five.

## Experiments and Results

The programs were developed in-house using MATLAB (Version 2015a; Natick, MA), and run on an IBM laptop with 3 GHz Intel i3 dual-processor and 8 GB random access memory (RAM).

### Key-slice selection

**Figure 6:**
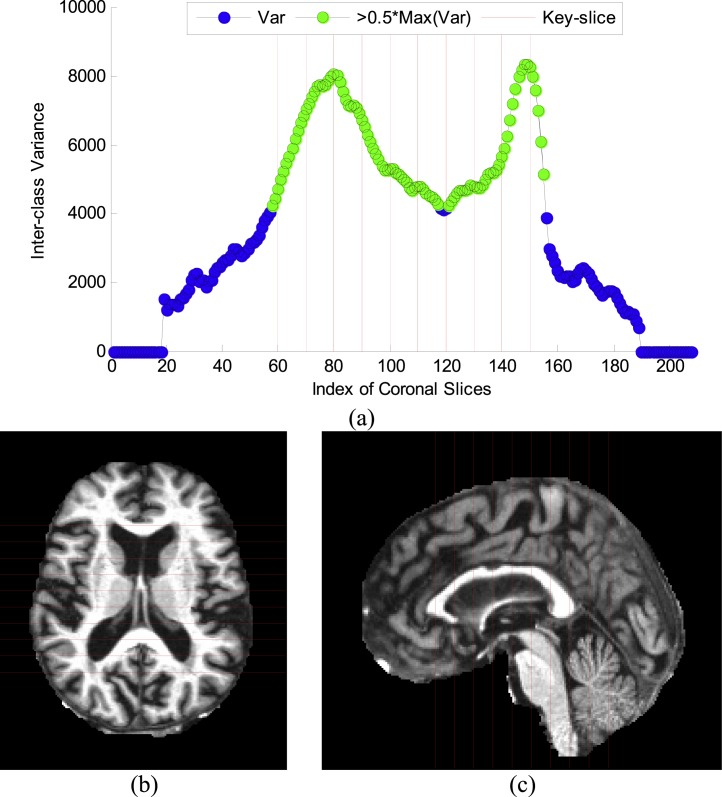
Results of key-slice selection. (A) Curve of ICV against CI. (B) Key-slices from axial view. (C) Key-slices from saggital view.

The curve of “inter-class variance” versus coronal slice index is shown in Fig. 6A. Ten coronal slices, from 60 to 150 with increasing steps of 10, were selected. The reason is that their corresponding ICVs were all higher than half of the maximum value. Figures 6B–6C shows respectively the axial and sagittal view of the selected key-slices, where the red lines represent the key-slices. The results are coherent with our formal work (Dong et al., 2015b).

### Displacement field

**Figure 7:**
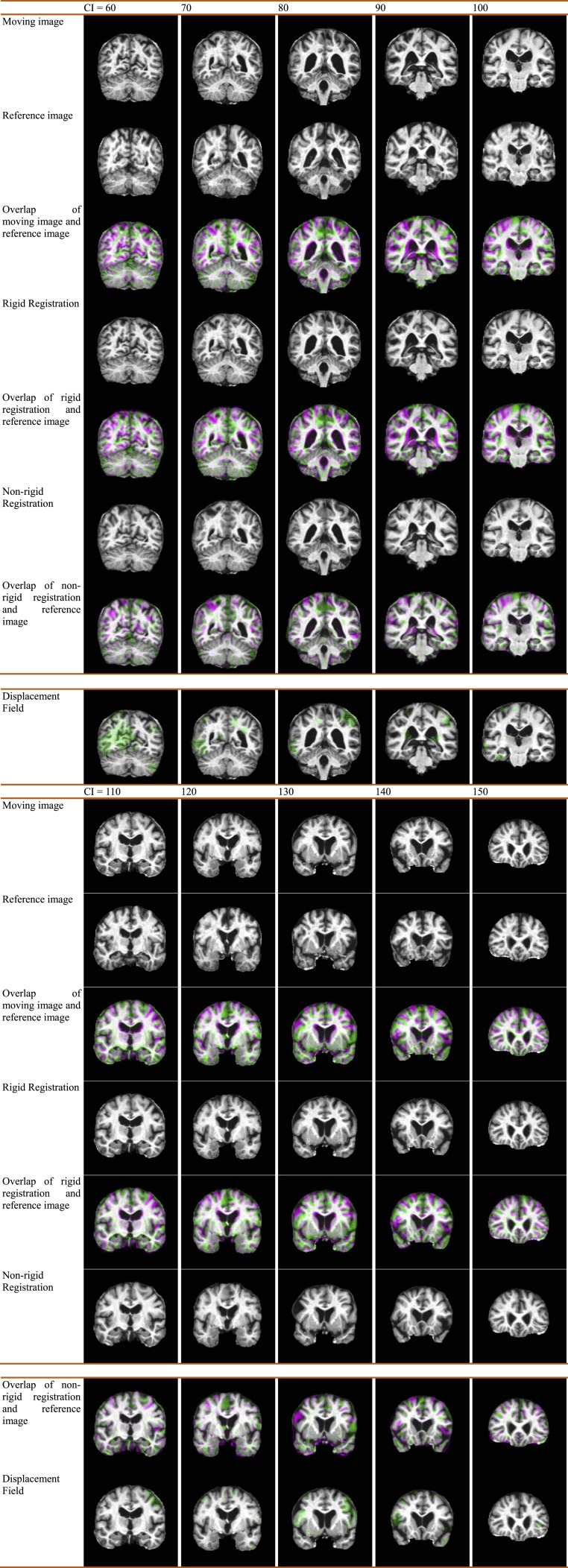
Displacement field of an AD with Coronal Index (CI). CI varies from 60 to 150 with increase of 10. (Please zoom in to see the displacement field.)

Figure 7 shows the displacement field of an AD over the key-slices. Each column corresponds to a key slice. Here CI means coronal index. We show the moving image, the reference image, the overlap of both, the rigid registration, the overlap of rigid registration and the reference image, the non-rigid registration, the overlap of non-rigid registration and the reference image, and the displacement field for each key slice. From the row of displacement field, we can see how that a healthy brain is warped into an AD brain through the displacement fields (green arrows in the bottom row).

### Classification comparison

The displacement fields were transformed into polar coordinate system, and their number was reduced by PCA as mentioned in the methodology section. Three classifiers were used: SVM, GEPSVM, and TSVM. Classification comparison results are provided in Table 5, together with the results of ten state-of-the-art methods.

**Table 5:** Comparison of different methods.

Existing methods	Accuracy	Sensitivity	Specificity	Precision
BRC + IG + SVM (Plant et al., 2010)	90.00 [77.41, 96.26]	96.88 [82.01, 99.84]	77.78 [51.92, 92.63]	N/A
BRC + IG + Bayes (Plant et al., 2010)	92.00 [79.89, 97.41]	93.75 [77.78, 98.27]	88.89 [63.93, 98.05]	N/A
BRC + IG + VFI (Plant et al., 2010)	78.00 [63.67, 88.01]	65.63 [46.78, 80.83]	100.00 [78.12, 100]	N/A
MGM + PEC + SVM (Savio & Grana, 2013)	92.07 ± 1.12	86.67 ± 4.71	N/A	95.83 ± 5.89
GEODAN + BD + SVM (Savio & Grana, 2013)	92.09 ± 2.60	80.00 ± 4.00	N/A	88.09 ± 5.33
TJM + WTT + SVM (Savio & Grana, 2013)	92.83 ± 0.91	86.33 ± 3.73	N/A	85.62 ± 0.85
US + SVD-PCA + SVM-DT (Wang et al., 2014)	90	94	71	N/A
EB + WTT + SVM (Dong et al., 2015b)	91.47 ± 1.02	90.17 ± 1.66	91.84 ± 1.09	75.93 ± 2.43
EB + WTT + RBF-KSVM (Dong et al., 2015b)	86.71 ± 1.93	85.71 ± 1.91	86.99 ± 2.30	66.12 ± 4.16
EB + WTT + POL-KSVM (Dong et al., 2015b)	92.36 ± 0.94	83.48 ± 3.27	94.90 ± 1.09	82.28 ± 2.78

Proposed methods	Accuracy	Sensitivity	Specificity	Precision
DF + PCA + SVM	88.27 ± 1.89	84.93 ± 1.21	89.21 ± 1.63	69.30 ± 1.91
DF + PCA + GEPSVM	91.52 ± 1.63	88.93 ± 1.80	92.27 ± 1.79	76.66 ± 2.33
DF + PCA + TSVM	92.75 ± 1.77	90.56 ± 1.15	93.37 ± 2.05	79.61 ± 2.21

### Region detection

**Figure 8:**
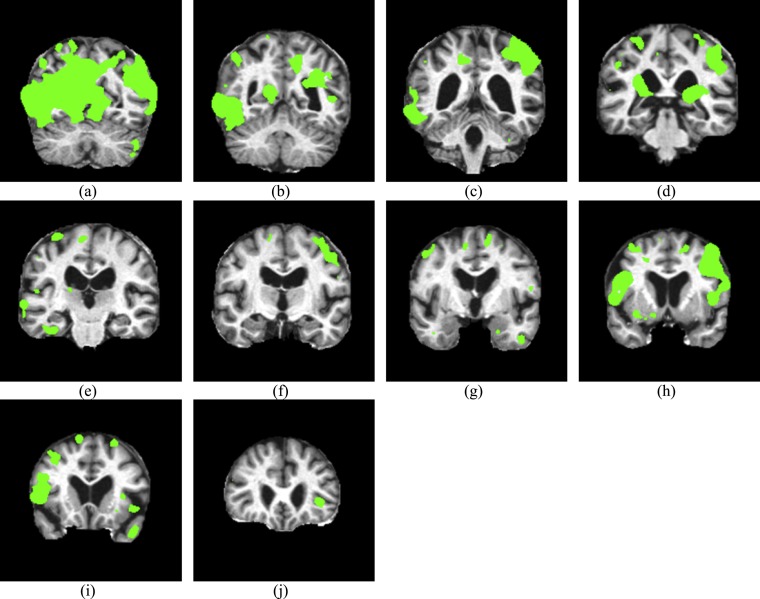
Related regions of AD. (A) CI = 60, (B) CI = 70, (C) CI = 80, (D) CI = 90, (E) CI = 100, (F) CI = 110, (G) CI = 120, (H) CI = 130, (I) CI = 140, (J) CI = 150.

We implemented the AD-related region detection procedure to different AD subjects as ‘Method of region detection’ described. Figure 8 shows the related regions, which are labelled by green points. Some areas are slightly outside of the brain, such as Fig. 8A, this is because the algorithm may also consider the distortion of background. This does not influence following labelling procedure.

### Area labeling

Talairach Daemon software was used to provide the anatomical label information. Table 6 shows the results, where BA represented Brodmann area. Note that some areas (such as cerebral tonsil, declive, and pyramis) move due to the expansion and shrinkage of neighboring areas, so they are not included in Table 6. We only pick up the areas with changed volume, which are judged manually.

**Table 6:** Discriminant areas with changed volume found by Talairach Daemon software.

Regions	# of voxels	Reported by
Angular gyrus	33	Carbonell et al. (2014)
Anterior cingulate (BA-33, BA-32, BA-24)	81	Schultz et al. (2014)
Cingulate gyrus (BA-32, BA-23, BA-24, BA-31)	1,551	Yu et al. (2014)
Culmen	396	Almeida et al. (2011)
Cuneus (BA-18, BA-30)	143	Yokoyama et al. (2015)
Fusiform gyrus (BA-18, BA-19, BA-20, BA-37)	314	Rieck et al. (2015)
Inferior frontal gyrus (BA-13, BA-45, BA-47)	320	Eliasova et al. (2014)
Inferior occipital gyrus	24	Liu et al. (2012)
Inferior parietal lobule (BA-2, BA-40)	311	Tramutola et al. (2015)
Inferior semi-lunar lobule	144	Almeida et al. (2011)
Inferior temporal gyrus (BA-20)	58	Narayan et al. (2015)
Insula (BA-44, BA-13)	328	Fletcher et al. (2015)
Lateral ventricle	33	Gonzalez-Marrero et al. (2015)
Lingual gyrus (BA-18, BA-19)	184	Cooley et al. (2015)
Medial frontal gyrus (BA-6, BA-32)	53	Jensen et al. (2015)
Middle frontal gyrus (BA-6, BA-46)	144	Yoo et al. (2015)
Middle occipital gyrus (BA-19)	175	Cai et al. (2015)
Middle temporal gyrus (BA-19, BA-20, BA-21, BA-22, BA-37, BA-38, BA-39)	485	Han et al. (2015)
Paracentral lobule (BA-5, BA-31)	161	Kang et al. (2013)
Parahippocampal gyrus (HC, BA-19, BA-30, BA-37)	62	Shimoda et al. (2015)
Postcentral gyrus (BA-2, BA-3)	244	Willette et al. (2015)
Posterior cingulate (BA-23, BA-30)	323	Pekarya, Sattin & Lloyd (2015)
Precentral gyrus (BA-4, BA-6, BA-13, BA-43, BA-44)	627	Wang et al. (2015c)
Precuneus (BA-7, BA-19, BA-31)	530	Villeneuve et al. (2015)
Sub-Gyral (Corpus, Callosum, BA-4, BA-13)	2,358	Ouyang et al. (2014)
Superior parietal lobule	21	Yamashita et al. (2014)
Superior temporal gyrus (BA-13, BA-22, BA-38, BA-39, BA-41)	462	Ramos et al. (2015)
Supramarginal gyrus (BA-40)	135	Redolfi et al. (2015)
Uncus (BA-20, BA-28, BA-34, BA-36, BA-38)	112	Bangen et al. (2014)

## Discussion

The results in Table 5 compare the proposed three classifiers (SVM, GEPSVM, and TSVM) with ten state-of-the-art methods. Plant’s results (Task 1 in Table 3 in Plant et al., 2010) presented the means together with 95% confidence intervals. Wang’s results (Table 7 in Wang et al., 2014) were obtained through a single K-fold cross validation analysis. Savio’s (Table 5 in Savio & Grana, 2013) and Dong’s results (Table 9 in Dong et al., 2015b) gave the means with SD.

Among the proposed methods, the proposed “DF + PCA + TSVM” yields the accuracy of 92.75 ± 1.77, sensitivity of 90.56 ± 1.15, specificity of 93.37 ± 2.05, and precision of 79.61 ± 2.21. Additional to it, the proposed “DF + PCA + GEPSVM” offers the accuracy of 91.52 ± 1.63, sensitivity of 88.93 ± 1.80, specificity of 92.27 ± 1.79, and precision of 76.66 ± 2.33. The proposed “DF + PCA + SVM” obtains the accuracy of 88.27 ± 1.89, sensitivity of 84.93 ± 1.21, specificity of 89.21 ± 1.63, and precision of 69.30 ± 1.91.

In terms of average accuracy, the proposed “DF + PCA + TSVM” result is as large as 92.75%, better than nine approaches of AD prediction, e.g., BRC + IG + SVM of 90.00% (Plant et al., 2010), BRC + IG + Bayes of 92.00% (Plant et al., 2010), BRC + IG + VFI of 78.00% (Plant et al., 2010), MGM + PEC + SVM of 92.07% (Savio & Grana, 2013), GEODAN + BD + SVM of 92.09% (Savio & Grana, 2013), US + SVD-PCA + SVM-DT of 90% (Wang et al., 2014), EB + WTT + SVM of 91.47% (Dong et al., 2015b), EB + WTT + RBF-KSVM of 86.71% (Dong et al., 2015b), and EB + WTT + POL-KSVM of 92.36% (Dong et al., 2015b). Nevertheless, the average accuracy of the method “DF + PCA + TSVM” is only less than one approach of “TJM + WTT + SVM” of 92.83% (Savio & Grana, 2013).

Why TSVM is better? There are two reasons. First, the non-parallel support vector machines provide more flexible and complicated hyperplanes than standard support vector machine. Second, the twin support vector machines formulate each of the two QP problems as a standard support vector machine, which makes it superior to generalized eigenvalue proximal support vector machine.

There were many other methods (Arbizu et al., 2013; Chaves et al., 2013; Cohen & Klunk, 2014; Dukart et al., 2013; Gray et al., 2012) proposed for detecting AD from NC, however, they dealt with images produced by other types of modalities: PET, SPECT, DTI, etc. Hence, it is inappropriate to compare the proposed methods with them. We will test our methods on SPECT and PET images in the future.

Table 6 shows that the displacement field finds the discriminant associated with the following regions reported in latest references: Angular Gyrus (Carbonell et al., 2014), Anterior Cingulate (Schultz et al., 2014), Cingulate Gyrus (Yu et al., 2014), Culmen (Almeida et al., 2011), Cuneus (Yokoyama et al., 2015), Fusiform Gyrus (Rieck et al., 2015), Inferior Frontal Gyrus (Eliasova et al., 2014), Inferior Occipital Gyrus (Liu et al., 2012), Inferior Parietal Lobule (Tramutola et al., 2015), Inferior Semi-Lunar Lobule (Almeida et al., 2011), Inferior Temporal Gyrus (Narayan et al., 2015), Insula (Fletcher et al., 2015), Lateral Ventricle (Gonzalez-Marrero et al., 2015), Lingual Gyrus (Cooley et al., 2015), Medial Frontal Gyrus (Jensen et al., 2015), Middle Frontal Gyrus (Yoo et al., 2015), Middle Occipital Gyrus (Cai et al., 2015), Middle Temporal Gyrus (Han et al., 2015), Paracentral Lobule (Kang et al., 2013), Parahippocampal Gyrus (Shimoda et al., 2015), Postcentral Gyrus (Willette et al., 2015), Posterior Cingulate (Pekarya, Sattin & Lloyd, 2015), Precentral Gyrus (Wang et al., 2015c), Precuneus (Villeneuve et al., 2015), Sub-Gyral (Ouyang et al., 2014), Superior Parietal Lobule (Yamashita et al., 2014), Superior Temporal Gyrus (Ramos et al., 2015), Supramarginal Gyrus (Redolfi et al., 2015), Uncus (Bangen et al., 2014).

**Table 7:** List of acronyms.

Acronym	Definition
(k) (GEP) (T) SVM	(kernel) (generalized eigenvalue problem) (twin) support vector machine
AD	Alzheimer’s disease
ANN	Artificial neural network
BA	Brodmann area
BD	Bhattacharyya distance
BRC	Brain region cluster
CAD	Computer-aided diagnosis
CC	Cerebral cortex
CDR	Clinical dementia rating
CSF	Cerebrospinal fluid
CI	Coronal index
CV	Cross validation
DBM	Deformation-based morphometry
DF	Displacement field
DWT	Discrete wavelet transform
GARCH	Generalized autoregressive conditional heteroscedasticity
GEODAN	Geodesic anisotropy
HC	Hippocampus
ICV	Inter-Class variance
IG	Information gain
KNN	K-nearest neighbors
MGM	Modulated GM
MMSE	Mini-mental state examination
MR(I)	Magnetic resonance (imaging)
NBC	Naive Bayes classifier
NC	Normal elder controls
OASIS	Open access series of imaging studies
PEC	Pearson’s correlation
PNN	Probabilistic neural network
PSO	Particle swarm optimization
QP	Quadratic programming
ROI	Region of interest
RQ	Rayleigh quotient
SD	Standard deviation
SVD	Singular value decomposition
TJM	Trace of Jacobian matrix
US	Undersampling
VFI	Voting feature intervals
WTT	Welch’s *t*-test

Notwithstanding, some regions reported to be associated with AD are not interpreted by displacement field. Those areas include caudate nucleus (Montagne et al., 2015), claustrum (Pirone et al., 2015), lentiform nucleus (Dong et al., 2015b), subcallosal gyrus (Dong et al., 2015b), and subthalamic nucleus (De Reuck et al., 2014). Why are those areas not detected by our DF method? The reasons are tri-fold: (1) We only preserved the displacement field with magnitude longer than 5. Reducing the value of 5 may include more potential regions and noises. A feasible solution is to reduce from 5 to 3 and meanwhile develop robust anti-noise method. (2) Some literature used advanced imaging modalities, such as MRSI and fMRI for metabolism detection and function analysis. Therefore, we may also include these imaging-modality techniques. (3) The key-slice selection procedure may miss important regions. Hence, we will try to reduce the slice separation, although it will increase the computation burden.

The **advantages** of displacement-field are two-fold. On one hand, it reaches excellent classification performance, which was comparable to latest approaches (Dong et al., 2015b; Plant et al., 2010; Savio & Grana, 2013; Wang et al., 2014). On the other hand, it can directly locate AD-related regions. The **disadvantages** of displacement field are: (i) The accuracy of displacement field estimation relies on the accuracy of rigid registration, which is a necessary pre-processing (see Fig. 3). (ii) The estimated DF may exist in the background (see Fig. 8A), which needs to be removed by masking technique.

## Conclusions

The **contributions** of this study consist of four points: (i) We proposed to use displacement field in the application of Alzheimer’s disease detection, and we proved its effectiveness. (ii) SVM and its two variants (GEPSVM and TSVM) were tested, and we proved TSVM performed better than SVM and GEPSVM. (iii) The proposed system achieved comparable sensitivity, specificity, and accuracy with ten state-of-the-art algorithms. (iv) The proposed computer-aided diagnosis (CAD) system can locate AD-related regions in the brain, which complies with 28 recent peer-reviewed articles.

**Future** research direction shall center in following aspects. First, the displacement field will be generalized to 3D, so key-slice selection can be discarded. Second, we shall try to embed kernels to classifiers. Third, spectroscopy method, like extracting the quantity of Docosahexaenoic Acid (DHA) (Tan et al.), may help to increase the classification accuracy. Forth, the proposed approach might be helpful in identifying brain mechanisms underlying endogenously defensive mechanism to neuroinjury and neurodegeneration (Yuan & Hou, 2015). Fifth, the least square technique (Hamedi, Salleh & Noor, 2015) may be applied to SVM and its variants, in order to further reduce training time. Sixth, the swarm-intelligence algorithm will be introduced to help to enhance the algorithm performance (Wang et al., 2015b). Finally, the stress field calculates the stress at every point within a particular region (Petit et al., 2015). It will be tested in future research.

## Supplemental Information

10.7717/peerj.1251/supp-1 Supplemental Information 1 A MRI Scan of AD

## Additional Information and Declarations

### Data Availability

The following information was supplied regarding data availability: Open Access Series of Imaging Studies (OASIS): http://www.oasis-brains.org/.
